# Recessive Resistance Derived from Tomato cv. Tyking-Limits Drastically the Spread of Tomato Yellow Leaf Curl Virus

**DOI:** 10.3390/v7052518

**Published:** 2015-05-21

**Authors:** Rita C. Pereira-Carvalho, Juan A. Díaz-Pendón, Maria Esther N. Fonseca, Leonardo S. Boiteux, Rafael Fernández-Muñoz, Enrique Moriones, Renato O. Resende

**Affiliations:** 1 National Center for Vegetable Crops Research (CNPH), Embrapa Hortaliças, CP 218, 70359-970 Brasília-DF, Brazil; E-Mails: carvalhorcp@gmail.com (R.C.P.-C.); maria.boiteux@embrapa.br (M.E.N.F.); leonardo.boiteux@embrapa.br (L.S.B.); 2 Department of Plant Pathology, University of Brasília (UnB), 70910-900 Brasília-DF, Brazil; 3 Institute for Mediterranean and Subtropical Horticulturev “La Mayora”, Málaga University—Spanish National Reseach Council (IHSM-UMA-CSIC), “La Mayora” Experimental Station, 29750 Algarrobo-Costa, Málaga, Spain; E-Mails: diazpendon@eelm.csic.es (J.A.D.-P.); rfern@eelm.csic.es (R.F.-M.); 4 Department of Cell Biology, University of Brasília (UnB), 70910-900 Brasília-DF, Brazil; E-Mail: rresende@unb.b

**Keywords:** *Begomovirus*, *Bemisia tabaci*, epidemiology, virus resistance, *Solanum lycopersicum*, TYLCV

## Abstract

The tomato yellow leaf curl disease (TYLCD) causes severe damage to tomato (*Solanum lycopersicum* L.) crops throughout tropical and subtropical regions of the world. TYLCD is associated with a complex of single-stranded circular DNA plant viruses of the genus *Begomovirus* (family *Geminiviridae*) transmitted by the whitefy *Bemisia tabaci* Gennadius (*Hemiptera*: *Aleyrodidae*). The tomato inbred line TX 468-RG is a source of monogenic recessive resistance to begomoviruses derived from the hybrid cv. Tyking F_1_. A detailed analysis of this germplasm source against tomato yellow leaf curl virus-Israel (TYLCV-IL), a widespread TYLCD-associated virus, showed a significant restriction to systemic virus accumulation even under continuous virus supply. The resistance was effective in limiting the onset of TYLCV-IL in tomato, as significantly lower primary spread of the virus occurred in resistant plants. Also, even if a limited number of resistant plants could result infected, they were less efficient virus sources for secondary spread owing to the impaired TYLCV-IL accumulation. Therefore, the incorporation of this resistance into breeding programs might help TYLCD management by drastically limiting TYLCV-IL spread.

## 1. Introduction

The tomato yellow leaf curl disease (TYLCD) is one of the major yield-limiting factors of tomato (*Solanum lycopersicum* L.) crops in tropical and subtropical regions [1,2]. This devastating disease is caused by a complex of single-stranded circular DNA plant viruses of the genus *Begomovirus* (family *Geminiviridae*) that are transmitted in a persistent circulative manner [3] by whitefly (*Hemiptera*: *Aleyrodidae*) members of the *Bemisia tabaci* Gennadius cryptic species. Among them, the monopartite tomato yellow leaf curl virus (TYLCV) is the most widespread and economically important worldwide [4]. Tomato plants affected by TYLCD exhibit characteristic symptoms of stunting, yellowing, upward curling of leaves, and suffer premature dropping of flowers and reduction of marketable fruits that can result in 100% yield loss when infections occur during early growth stages [1,5].

The control of TYLCD is often based on intensive chemical treatments to limit vector population and/or by using physical barriers, both with limited success. In addition, chemical control result in deleterious environmental effects and can determine selection of insecticide-resistant *B. tabaci* populations [6,7]. Therefore, in this scenario, the use of resistant tomato cultivars is the most environmentally sustainable and economically viable approach to reduce TYLCD damage. 

Susceptibility to TYLCV requires virus replication in the plant cell nucleus via a double-stranded DNA intermediate, movement to adjacent cells through plasmodesmata, long-distance movement through the phloem, and further acquisition by vectors for transmission from plant to plant to reinitiate the infection cycle. A block at any of these steps, either by active defense responses or by incompatible interactions of viral and host factors, may lead to virus resistance [8]. A number of virus resistance factors have been derived from wild tomato relative species that can help restricting TYLCD-associated virus infection and limit disease damage [9]. The partially dominant *Ty*-1 resistance gene derived from the *S. chilense* accession LA1969 [10] is so far the most widely used commercially. However, the performance of *Ty*-1 or other plant host resistance genes varies depending upon the TYLCD-causing virus [11,12]. Also, breakdown of *Ty*-1 resistance can occur under high inoculum pressure [13] and it often shows lower effectiveness in heterozygous plants used commercially [14]. Furthermore, emergence of recombinant TYLCD-viruses with novel pathogenic characteristics [15,16] might pose a threat to available resistance genes. Recently, we reported an alternative tomato source of begomovirus resistance named TX 468-RG, which was derived via selfing from the commercial F_1_ hybrid “Tyking” (released by Royal Sluis, The Netherlands). This inbred line displayed high levels of resistance to bipartite and monopartite begomoviruses associated to TYLCD based on single recessive gene control [17,18]. A previous work allowed us to clarify the genetic control of the TX 468-RG resistance and to demonstrate its effectiveness to a range of TYLCD-associated monopartite begomoviruses [17]. However, the resistance mechanism remains unsolved. In the present study, a detailed set of analyses was conducted to understand the restriction to accumulation of an isolate of the Israel strain of TYLCV (TYLCV-IL) in TX 468-RG and to evaluate to what extent this resistance can help to limit virus spread under field conditions. We conclude that although no effect in inoculated leaves was observed, systemic infection of TYLCV-IL was impaired in TX 468-RG plants, resulting in reduced virus accumulation. As a consequence, TX 468-RG resistance was effective to limit primary and secondary spread of the virus. Therefore, this resistance is highly recommended for breeding purposes to control damage caused by TYLCV-IL in tomato.

## 2. Materials and Methods

### 2.1. Tomato Plants, Virus Isolate, and Whitefly Population

A tomato F_8_ inbred line (named as “TX 468-RG”) was derived via repeated selfing and selection steps from the commercial F_1_ hybrid “Tyking” (released in the 1990s by Royal Sluis, The Netherlands) and used in all assays. This germplasm displayed high levels of resistance to bipartite begomoviruses [18] as well as to a range of TYLCD-associated monopartite begomoviruses [17]. The open-pollinated tomato cv. Moneymaker (MM) (IHSM-UMA-CSIC seed bank) was used as susceptible control in the experiments.

The infectious clone of the isolate [ES:Alm:Pep:99] of TYLCV-IL (TYLCV-IL [ES:Alm:Pep:99], from now on, TYLCV-IL) (GenBank accession number AJ489258), has been described elsewhere [19]. Healthy *B. tabaci* adult individuals were obtained from a colony of the Mediterranean (MED) species (formerly known as Q biotype) originated from field individuals collected in Malaga, Spain. Whiteflies were reared on melon (*Cucumis melo* L. cv. ANC42, IHSM-UMA-CSIC seed bank) plants within wooden cages covered with insect-proof nets, in an insect-proof glasshouse with temperature control (22–27 °C day and 17–20 °C night) and supplemental light when needed. 

### 2.2. Virus Inoculation

*Agrobacterium tumefaciens-*mediated stem puncture inoculation (agroinjection) with TYLCV-IL was conducted on plants at the three-leaf growth stage as described previously [20]. Also, for TYLCV-IL local accumulation studies, *A. tumefaciens-*mediated leaf tissue infiltration (agroinfiltration) studies were conducted following Tomás *et al.* [21]. Either for agroinjection or for agroinfiltration, bacterial suspensions at OD_600_ = 1.0 were used. For *B. tabaci*-mediated inoculation, viruliferous whiteflies were obtained by providing insect adults with a 48 h acquisition access period (AAP) on systemically infected young leaves of MM plants agroinjected with TYLCV-IL three weeks earlier. 

For graft inoculation, healthy scions of TX 468-RG and MM were grafted onto MM and TX 468-RG plants previously infected with TYLCV-IL by agroinjection. Scions consisted of a stem piece containing a leaf with its associated lateral shoot meristem obtained from healthy test plants. Grafts of healthy MM or TX 468-RG scions were made on both MM and TX 468-RG infected rootstocks by splice-grafting. The grafted plants were then kept in a shaded and humid environment within a growth chamber for seven days and then moved to the insect-proof glasshouse (see below) until evaluation. Evaluations of TYLCD symptom and presence/accumulation of TYLCV-IL in young developing leaves of the scions was done at 28 days post-grafting by visual observation and by tissue blot and dot blot hybridization analyses of individual plants, respectively.

Plant inoculations were performed in a growth chamber (25 °C day and 20 °C night, 70% relative humidity, with a 16 h photoperiod at 250 µmol·s^−1^·m^−2^ photosynthetically active radiation), and the inoculated plants were then kept until analyzed in an insect-proof glasshouse with temperature control (approximately 16 h day length, at 22 to 27 °C during the day and 17 to 20 °C at night) and supplemental light when needed.

### 2.3. Primary and Secondary TYLCV-IL Spread Experiments

Primary and secondary spread experiments were conducted at “La Mayora” Experimental Station (Malaga, southern coastal Spain) essentially as described by Rodríguez-López *et al.* [22]. Briefly, primary spread of TYLCV-IL, *i.e.*, virus spread to healthy plants from external source of viruliferous vectors [23], was simulated in medium-scale experiments conducted within insect-proof net, walk-in structures (5 × 5 × 2 m) built within a tunnel net house, by releasing adult viruliferous individuals (15 whiteflies per test plant) for a 48 h inoculation access period (IAP). Viruliferous whiteflies were placed in the center of a circle (2 m diameter) of 22 MM or TX 468-RG healthy test plants at the four-leaf growth stage in no-choice test design or of 11 healthy plants of each genotype placed in an alternate design in free choice test design (Supplementary Figure 1). After the IAP, plants were treated with insecticide and then transferred to an insect-proof glasshouse (see description above) until analysis. Secondary spread from TYLCV-IL-infected plants, *i.e.*, virus spread from virus-infected source plants to healthy plants [23], was simulated in similar medium scale experiments and insect-proof net walk-in structures. In this case, three TYLCV-IL-infected virus source plants were placed forming a triangle (with 60 cm between the three plants) in the center of a circle (2 m diameter) of 22 MM or TX 468-RG healthy test plants at the four-leaf growth stage in a no-choice test design (Supplementary Figure 1). Virus-source plants were MM or TX 468-RG plants agroinjected with TYLCV-IL 15 days earlier. Healthy *B. tabaci* adult individuals (30 whiteflies per test plant) were then released in the center of the triangle of virus-source plants, and after 96 h, test plants were treated with insecticide and transferred to an insect-proof glasshouse until analysis. Control treatments were included using healthy whiteflies for primary spread and healthy source plants for secondary spread with MM as test and source plants. Virus presence was scored in young newly grown leaves of test plants by tissue blot hybridization (see below) at weekly intervals until 28 dpi. Three independent primary and secondary virus spread experiments were conducted.

### 2.4. B. tabaci Preference

Preference of *B. tabaci* whiteflies for MM or TX 468-RG was studied in separate, no-choice experiments in which 50 plants of each genotype (plants at the four-leaf growth stage) were placed within a walk-in structure like those used in the virus spread experiments and at equivalent growth conditions. Healthy adult individuals (30 whiteflies per test plant) were released in the center of the arena and whiteflies visiting the plants were counted 24 h after release in three leaflets per plant, located at the lower, middle and upper parts of the plant. Number of whiteflies per plant corresponded to the sum of the three values obtained per plant. Distribution of values was represented by Box-and-Whisker plots [24]. 

### 2.5. Virus Detection and Determination of TYLCV-IL Accumulation Levels in Infected Tomato Plants

Virus presence was analyzed in young leaves of inoculated plants by tissue-blot hybridization of freshly cross-sectioned leaf petioles or by Southern blot hybridization of total nucleic acid (TNA), according to Tomás *et al.* [21] using a probe specific to TYLCV-IL. To monitor virus accumulation in young leaf tissues of TX 468-RG and MM plants inoculated with TYLC-IL, dot blot hybridization analyses were conducted. For this, TNA extracts were prepared from the second leaf from the apex, as described by Celix *et al.* [25], except that TNA were dissolved in 50 μL of sterile H_2_O in the final step. TNA extracts were quantified, the concentrations standardized at 200 ng/μL, and used for dot blot hybridization. For estimation of TYLCV-IL accumulation levels, different groups of inoculated plants were sampled in a destructive manner at 7, 14, 21, and 28 days post inoculation (dpi) (about 14 plants per evaluation date). Two microliter per sample of each dilution of a dilution series 1:1, 1:2, 1:4, 1:8, 1:16, 1:32, 1:64, 1:128, 1:256, and 1:512 of TNA were applied to positively charged nylon membranes (Roche Diagnostics GmbH, Mannheim, Germany). Two replicated membranes were prepared for DIG-labeled DNA-probe hybridization, one for hybridization with a probe to TYLCV [26] and the other with a probe to a gene fragment coding for 18S ribosomal RNA (18S rRNA) [27], as loading control. Healthy MM plants were used as negative controls. Assessment of TYCLV-IL DNA content was done by densitometric measure of hybridization signals obtained in autoradiographs. Plant ribosomal RNA signals were used as an internal standard to equilibrate TNA loading among samples. Densitometry measurements were expressed in pixels measured using Quantity One Software v 4.6.7 (VersaDoc MP 4000 Imaging System; BioRad), giving an arbitrary value of 1000 to that of one of the MM infected plants per date analyzed, and referring the other values to this one. In every case, the densitometry values used for estimation of TYLCV-IL accumulation levels were those from a dilution that fell within the linear range of the relationship between dilution and densitometry measure. Distribution of values was represented by Box-and-Whisker plots [24]. 

### 2.6. Data Analysis

Statistical effects of genotypes and/or treatments in the different experiments were analyzed with the IBM SPSS Statistics v. 22 software by applying Generalized Linear Models (GzLM), in which all possible pair-wise comparisons were performed using the sequential Bonferroni method for error correction. For the case of comparisons of the TYLCV-IL accumulation levels estimated, the GzLM used Logarithm as the link function and Normal as the underlying distribution. For TYLCV incidence in the primary and secondary spread experiments, data set was expressed as the number of infected and non-infected plants at each time point and were analyzed by GzLM using Logit as the link function and Binomial as the underlying distribution. Also, a general disease incidence pressure was estimated for combinations of genotypes and treatments in those experiments by calculating the Area Under the Disease Progression Curve (AUDPC) using the formula AUDPC = Σ((Yi + Y(i+1))(T(i+1) – Ti))/2, where Yi = proportion of infected plants at date i, and Ti = time (in days) at date i. For comparing means of AUDPC values, the GzLM used Identity as the link function and Normal as the underlying distribution. Finally, for comparison of the number of whiteflies visiting the two tomato genotypes in the preference experiment, whiteflies per plant data were analyzed by GzLM using Logarithm as the link function and Negative Binomial as the underlying distribution.

## 3. Results

### 3.1. TYLCV-IL Accumulation is Highly Restricted in TX 468-RG

**Figure 1:**
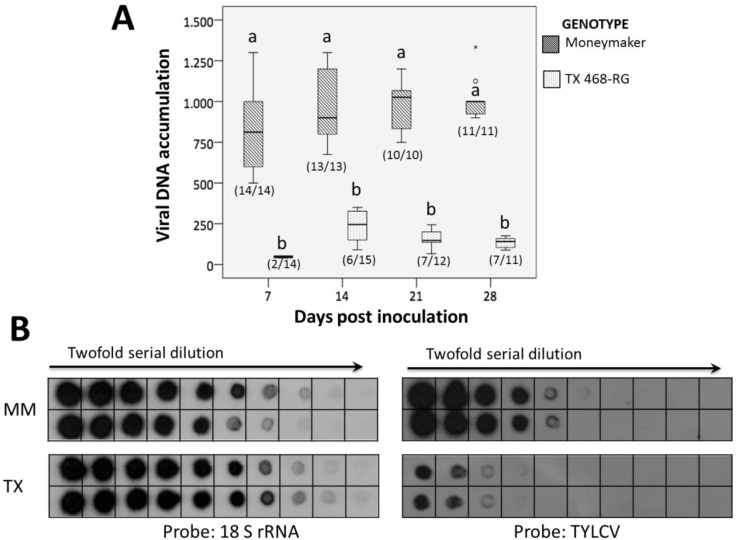
(**A**) Accumulation of tomato yellow leaf curl virus (TYLCV) DNA at several days post inoculation (dpi) in non-inoculated young leaves of systemically infected TX 468-RG and “Moneymaker” tomato plants inoculated with an isolate of the Israel strain of TYLCV (TYLCV-IL) by *Agrobacterium tumefaciens*-mediated inoculation. One independent group of plants was used per tomato genotype and date. Virus DNA content is estimated from densitometry measure of hybridization signals obtained in digitized imaging of autoradiographs (kilopixels) of dot blot hybridizations using a TYLCV-IL-specific probe and total nucleic acids (TNA) extracted from plants and normalization of values per plant with the signal from the hybridization with a 18S ribosomal RNA probe. Densitometry values corresponding to the dilution 1:1 of 400 ng of TNA are represented in a Box-and-Whisker plot. At each date, an arbitrary value of 1000 was given to the value of one MM infected plant and values of the other plants were referred to this one. In each case, the number of plants that resulted infected *vs.* the number of inoculated plants was indicated at the bottom of each box. Outliers are shown and at each date, values with the same letter are not significantly different (sequential Bonferroni tests under Generalized Linear Model, *p* = 0.05); (**B**) Dot blot hybridization of serial dilution 1:1, 1:2, 1:4, 1:8, 1:16, 1:32, 1:64, 1:128, 1:256, and 1:512 of TNA extracts from two TYLCV-IL-infected plants each of TX 468-RG (TX) and “Moneymaker” (MM) tomatoes at 28 dpi. Equivalent dot blot dilution series were analyzed with probes against to either TYLCV-IL or to 18S rRNA gene as loading control.

Systemic TYLCV-IL infection was observed in all inoculated MM plants, whereas lower incidence levels were observed in TX 468-RG plants, especially at initial evaluation dates (see number of plants infected *vs.* number of plants inoculated at the bottom of boxes, Figure 1A). Also, no symptoms were observed in any TX 468-RG-infected plant, whereas typical TYLCD symptoms were observed in all infected MM plant from 21 dpi. Interestingly, viral DNA accumulation in infected plants revealed a strong restriction in TX 468-RG. Although viral DNA could be detected in young tissues of a number of these plants, confirming that they are not immune (*sensu* [28]) to TYLCV-IL, significantly lower accumulation levels were observed (Figure 1A) when compared to plants of the susceptible MM control (see e.g., dot blot hybridization for two representative plants from the two genotypes at 28 dpi in Figure 1B). Thus, at 28 dpi, about eight times lower viral DNA accumulation was detected in young tissues of systemically infected TX 468-RG than in the equivalent ones of MM plants. 

**Figure 2:**
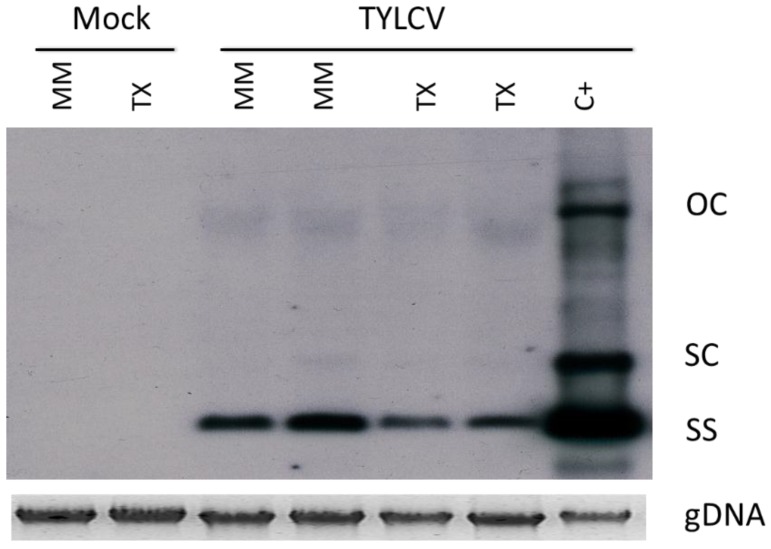
Southern blot hybridization analysis of total nucleic acids extracted from TX 468-RG (TX) and “Moneymaker” (MM) leaves agroinfiltrated with an infectious clone of an isolate of the Israel strain of tomato yellow leaf curl virus (TYLCV-IL). The analysis was conducted at five days post *Agrobacterium tumefaciens-*mediated infiltration. Equivalent preparations from noninfected (Mock) or from TYLCV-IL-systemically infected tissues (C+) of control tomato plants were included. Positions are indicated for the single-stranded genomic DNA (SS) and for the open circular (OC) and supercoiled double-stranded DNA forms of TYLCV-IL derived from input DNA; genomic DNA (gDNA) was included as loading control.

By contrast, no major differences in accumulation levels of viral forms derived from the input DNA was observed at local agroinfiltrated tissues of TX 468-RG and MM plants (Figure 2), suggesting that constraints at initial steps of viral infection does not seem to be the basis of the resistance mechanism. Therefore, differences in virus accumulation might occur during the systemic infection process.

**Figure 3:**
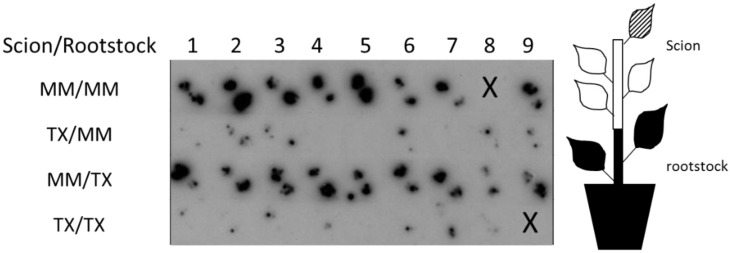
Detection of an isolate of the Israel strain of tomato yellow leaf curl virus (TYLCV-IL) in tissue blots of cross sections of petioles from newly emerged young leaves (two blots per sample were made) of TX 468-RG or “Moneymaker” healthy scions grafted onto “Moneymaker” or TX 468-RG rootstocks infected with TYLCV-IL. Detection was performed at 28 days after grafting by hybridization using a DIG-labeled DNA probe to TYLCV-IL. X indicates no sample present. A schematic representation of the grafted plant and the leaf analyzed (shadowed) is shown at the right of the figure.

To investigate whether virus resistance in TX 468-RG occurred during the process of systemic virus translocation, grafting of TX 468-RG and MM healthy scions onto TYLC-IL-infected rootstock were analyzed. Although tissue blot hybridization is not a quantitative assay, the results shown in Figure 3 strongly suggest that resistance in TX 468-RG impaired TYLC-IL accumulation during systemic translocation and was operative even under a continuous virus supply. Thus, only traces of virus were observed in all TX 468-RG scions either grafted onto MM or TX 468-RG rootstocks infected with TYLCV-IL, supporting that the level of virus accumulation in this genotype was not related to the amount of inoculum supplied. Also, no TYLCD symptoms were ever observed in any of the TX 468-RG grafted scions. In contrast, severe symptoms and higher virus accumulation was observed in MM scions grafted either onto MM or onto TX 468-RG rootstocks infected with TYLCV-IL, even if in the latter case low virus titers occurred in the rootstock (see Figure 1). Therefore, the begomovirus-resistance present in TX 468-RG was able to impair the TYLCV-IL systemic translocation within the plant even under high viral loads. 

### 3.2. TX 468-RG Results in a Reduced Primary Spread of TYLCV-IL

**Figure 4:**
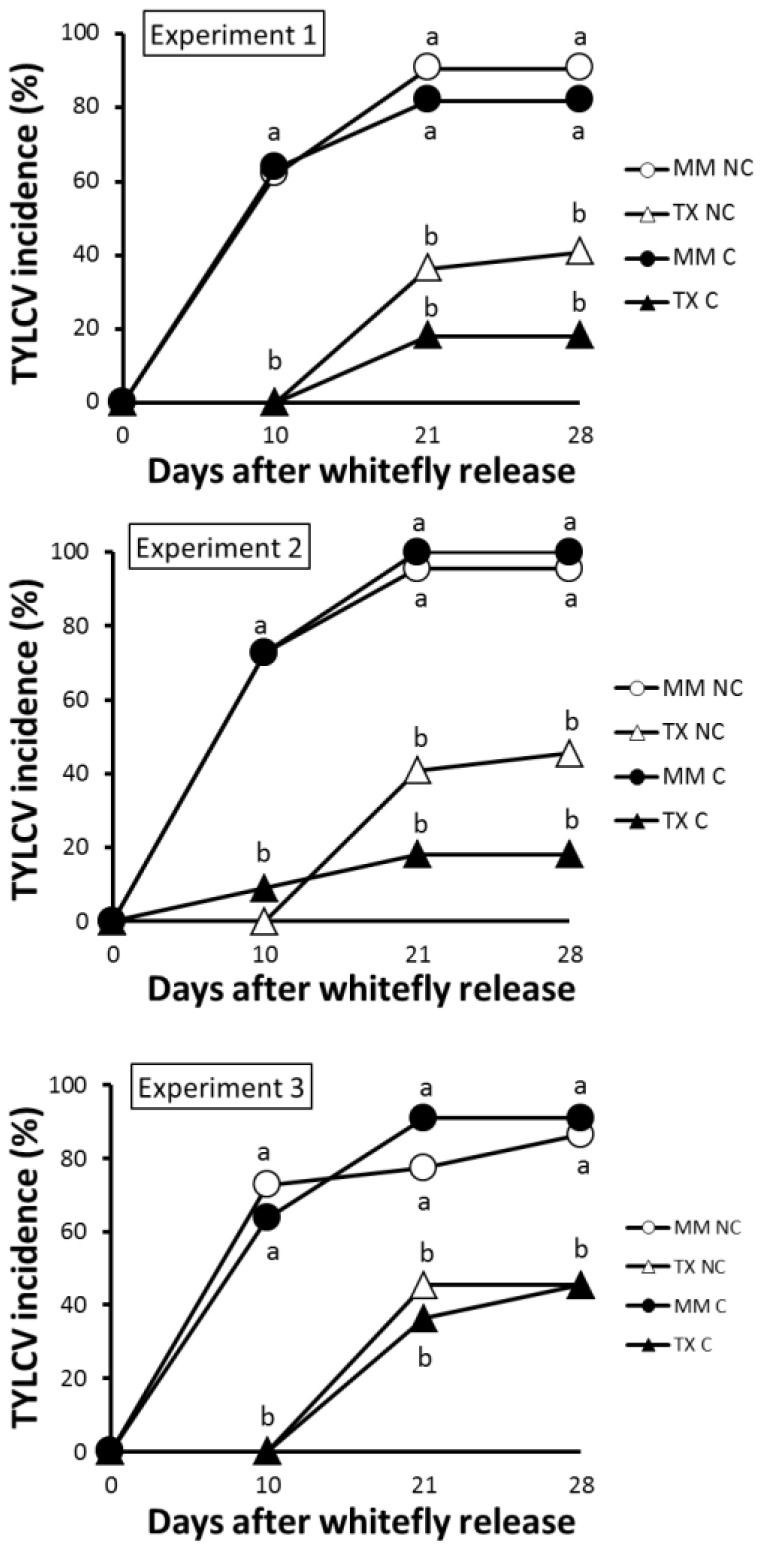
Fraction (%) of plants infected with an isolate of the Israel strain of tomato yellow leaf curl virus (TYLCV-IL) in experiments measuring primary virus spread to tomato plants under no-choice (NC) (22 TX 468-RG or “Moneymaker” healthy test plants) or free-choice (C) (11 TX 468-RG + 11 “Moneymaker” healthy test plants in an alternate design) conditions. Virus presence in young leaves of test plants was scored by tissue blot hybridization at several times after viruliferous whiteflies (15 *Bemisia tabaci* MED adult whiteflies per test plant) were given a 48 h feeding and inoculation access period. At each date, values with the same letter are not significantly different (sequential Bonferroni tests under a Binomial-Logistic Generalized Linear Model, *p* < 0.05). Results for the three independent experiments conducted are shown. Schematic representation of the experimental design can be seen in Supplementary Figure S1.

The results of the study conducted to estimate the effect of plant genotype on primary virus spread showed that significantly lower incidence levels were observed in TX 468-RG in relation to MM, either in choice or in no-choice conditions in all the three independent experiments conducted (Figure 4). Over 80% incidence was achieved at 28 dpi in MM, whereas about half of this value was observed for TX 468-RG. The resistant line also displayed a significant delay in the onset of the infection with a significantly reduced number of infected plants at 10 dpi. All this resulted in significantly lower AUDPC values for TX 468-RG when compared to MM (5.2 ± 0.3 *vs.* 18.3 ± 0.6 for no-choice and 2.7 ± 1.4 *vs.* 18.4 ± 0.9 for free-choice) and, therefore, lower disease pressure in TX 468-RG under our experimental conditions. Thus, the use of TX 468-RG-derived resistance would result in a significantly reduced primary spread of TYLCV-IL and lower virus pressure under field conditions.

### 3.3. TX 468-RG Limits Secondary Spread of TYLCV-IL from Infected Source Plants to either TX 468-RG or MM 

**Figure 5:**
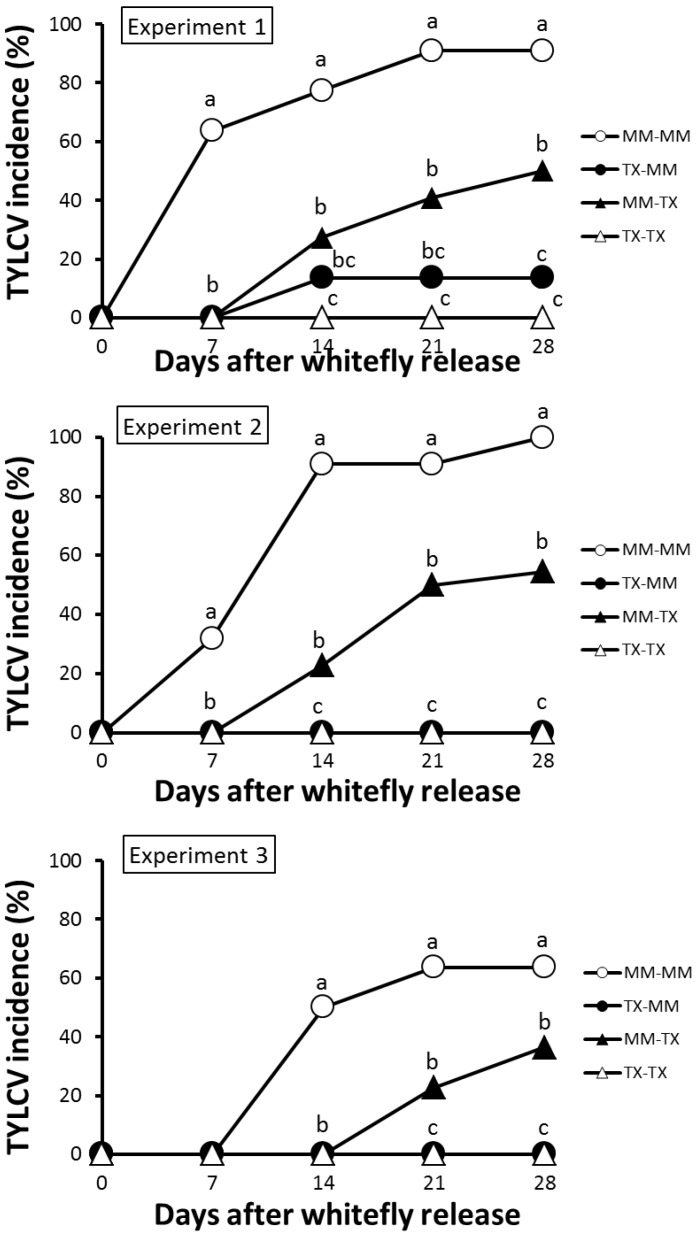
Fraction (%) of plants infected with an isolate of the Israel strain of tomato yellow leaf curl virus (TYLCV-IL) in experiments measuring secondary virus spread from TX 468-RG or “Moneymaker”-infected tomato plants to TX 468-RG or “Moneymaker” healthy tomato plants (22 test plants in a no-choice test). Virus presence in young leaves of test plants was scored by tissue blot hybridization at several times after nonviruliferous whiteflies (30 *Bemisia tabaci* MED adult whiteflies per test plant) were given a 96 h feeding and inoculation access period. At each date, values with the same letter are not significantly different (sequential Bonferroni tests under a Binomial-Logistic Generalized Linear Model, *p* < 0.05). Results for the three independent experiments conducted are shown. Schematic representation of the experimental design can be seen in Supplementary Figure S1.

Because the previous results indicated that TYLCV-IL could infect TX 468-RG plants during virus spread, secondary spread of TYLCV-IL to both TX 468-RG and MM healthy test plants was assessed. As summarized in Figure 5, the results of the three independent experiments conducted clearly showed that significantly lower secondary spread occurred when TX 468-RG was present, as source and/or test plant, with no infections observed when this genotype was used as source and test plant. Therefore, theTYLCV-IL resistance of TX 468-RG is strongly effective in reducing the secondary spread of this virus. As a result, a significantly lower infection pressure occurred in test plants (AUDPC 16.0 ± 2.9 for secondary spread from MM to MM *vs.* 5.5 ± 1.3, 0.8 ± 0.8, and 0, for secondary spread from TX 468-RG to MM, MM to TX 468-RG, and TX 468-RG to TX 468-RG, respectively). Therefore, lower TYLCD infection pressure under field conditions is expected when using this resistance.

### 3.4. B. tabaci Exhibited no Preference for either MM or TX 468-RG

**Figure 6:**
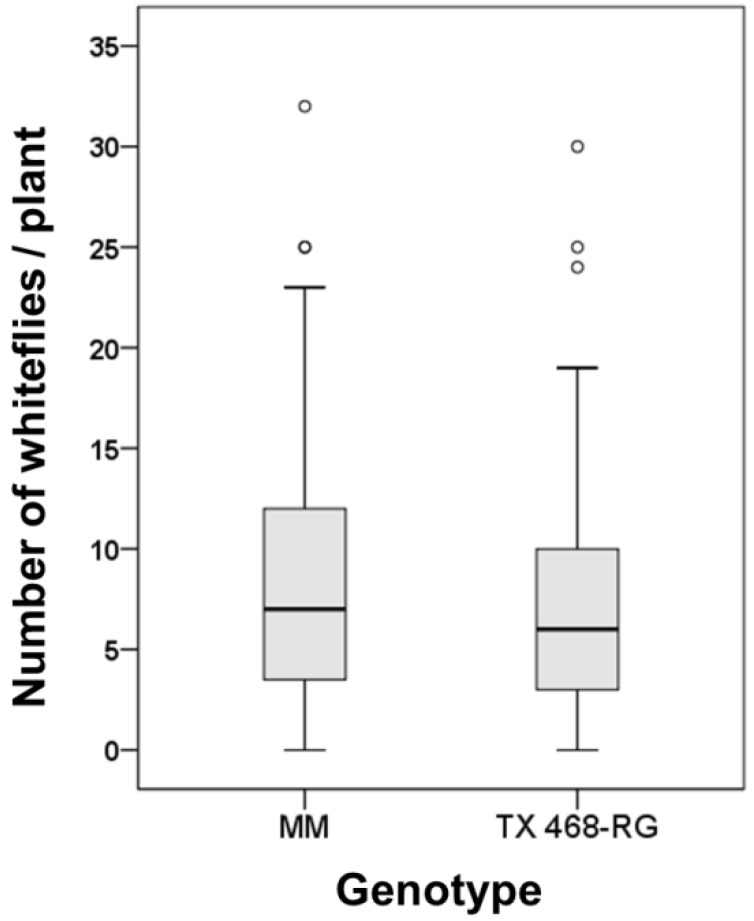
*Bemisia tabaci* settling on TX 468-RG or “Moneymaker” (MM) healthy tomato plants (50 test plants in a no-choice preference test). Number of whiteflies per test plant (sum of numbers present in three leaflets of each test plant) at 24 h after release of 30 *B. tabaci* MED adult whiteflies per test plant is represented in a Box-and-Whisker plot. Outliers are shown.

No significant difference in whitefly preference for either MM or TX 468-RG tomato genotypes was observed based on the data obtained from the no-choice preference experiment (MM, 9.5 ± 1.4 *vs.* TX 468-RG, 8.3 ± 1.3 whiteflies per plant; *p* = 0.553) (Figure 6). Therefore, results from spread experiments were not influenced by differences in vector behavior depending on the tomato genotype.

## 4. Discussion

The use of tomato cultivars with genetic resistance has been the most effective strategy to minimize losses caused by viral diseases, including pathosystems involving *Begomovirus* species [9]. The current study advances our understanding about the benefits of using virus resistant genotypes to limit TYLCV spread in the field. A compatible virus interaction with a plant involves effective multiplication and movement of the virus from the site of infection and throughout the plant as well as effective transmission to other plants to guarantee maintenance in nature [29]. However, during the virus/host plant co-evolution process, distinct host plant mechanisms to restrict virus infection can be selected and sources of resistance can become predominant in natural plant populations under continuous disease pressure due to their selective advantages [28]. Here, we demonstrated that the previously reported monogenic recessive resistance against TYLCV-IL present in TX 468-RG [17] strongly impairs systemic virus infection, resulting in a significantly reduced primary and secondary spread of the virus. Therefore, this resistance is proposed as a good and effective alternative for breeding purposes to reduce TYLCD damage in commercial tomatoes. 

Previous observations for the usefulness of TX 468-RG resistance to control TYLCV-IL [17] were confirmed by the detailed studies conducted here. This is important because TYLCV-IL is one of the most widespread TYLCD-associated virus worldwide [4] causing severe economic losses to tomato production where present. TX 468-RG plants, however, are not immune and can be infected with this virus. Nevertheless, we show here that a severe restriction to systemic virus accumulation occurred in infected plants harboring this recessive resistance. Leaf agroinfiltration experiments demonstrated that local accumulation of TYLCV-IL was not impaired in TX 468-RG plants, suggesting no effect on the initial steps of infection and that the restriction occurred during the systemic infection process [30]. Based on our studies, however, we cannot rule out whether the latter restriction is due to impairment of viral movement and/or to the triggering of plant defenses, such as gene silencing [31]; further research will be needed to address this issue. Similar restriction to systemic infection was shown to be conferred to another monopartite begomovirus, *Tomato leaf curl virus* by the recessive resistance *tgr*-1 gene present in the line FLA-653 [32], derived from a cross between the resistant genotypes “Tyking” and *S*. *chilense* LA2779. In this case, however, Bian *et al.* [32] observed that in addition to a limitation of long-distance translocation a restriction also occurred at local level, which was associated with impaired cell-to-cell movement of the virus. Restriction to systemic virus accumulation has also been reported for the cultivar “Tyking” when confronted by other begomoviruses [33]. Interestingly, we showed that similarly to that reported for the breeding tomato line TY172 resistant to TYLCV [34], the plant defense mechanism operating in TX 468-RG against TYLCV-IL is not overcome by continuous supply of high loads of virus. Therefore, contrary to that observed for the *Ty*-1 gene widely used commercially [13], effectiveness of the recessive resistance studied here is expected even under high disease pressure, in accordance with preliminary field studies [17]. 

Spread of TYLCD by whiteflies in the field generally occurs in two phases. In a first instance, onset of the disease in a crop occurs from external sources of inoculum, *i.e.*, primary spread, which usually leads to a random distribution pattern of primary infections foci [23]. Then, secondary spread from these primary sources of infection can occur within the crop, whose intensity will strongly depend on the magnitude of the insect vector population present in the crop. Here, we demonstrate that the resistance present in TX 468-RG was effective to severely limit primary spread of TYLCV-IL, thus resulting into a first barrier to reduce TYLCD field epidemics. As no significant difference on the *B. tabaci* preference was observed between TX 468-RG and MM, spread restriction was mainly based on the resistance to TYLCV-IL present in the former genotype. We observed, however, that a small number of TX 468-RG plants could get infected, even though no virus damage occurs in these plants due to the resistance factor [17]. These infected TX-468-RG plants constitute potential sources for secondary virus spread within the crop or even for primary spread to nearby susceptible crops. In fact, there are reports supporting the threat of some tomato genotypes resistant but not immune to TYLCV as efficient sources for secondary virus spread [35,36]. However, we demonstrate here that the level of resistance expressed by TX 468-RG was effective to drastically restrict secondary spread of the virus. This finding suggests that TX 468-RG plants restricted virus accumulation to levels that resulted in impaired insect transmission [37], and is compatible with the low transmission rates observed by Lapidot *et al.* [35] from tomato plants highly resistant to TYLCV. This is an important aspect for TYLCV management, as virus transmission from infected resistant plants can determine the field success of the resistant genotype to control TYLCD epidemics [35,36]. In conclusion, the resistance present in TX 468-RG was effective to reduce primary and secondary spread of TYLCV-IL. Therefore, this resistance can help in effective management of TYLCD under field conditions. 

Even though some TYLCD-associated virus-resistant cultivars are available commercially, owed to the resistance breakdown occasionally observed under high disease pressure [13], control measures traditionally have emphasized reducing vector populations through chemical control [38]. Concerns exist, however, about intensive use of insecticides due to the environmental damage caused and to development of pesticide resistance in insect population [6,7]. Therefore, based on the results shown here, the TYLCV-IL resistance present in TX 468-RG offers a good alternative for TYLCD-resistance breeding programs to incorporate this character into new hybrids and cultivars. Moreover, since TX 468-RG has proven to also be resistant to bipartite begomoviruses [18] with a similar reduction in viral accumulation, the same epidemiological impact on their spread is expected. Therefore, this source of resistance can be an important component of broad management of begomoviruses in tomato.

## Supplementary Files

Supplementary File 1
