# Supplementary material for: Recessive Resistance Derived from Tomato cv. Tyking-Limits Drastically the Spread of Tomato Yellow Leaf Curl Virus
Source: Viruses. 2015 May 21;7(5):2518–33. doi: 10.3390/v7052518 (PMC4452918; doi:10.3390/v7052518)
Supplement: Supplementary File 1 [file viruses-07-02518-s001.pdf]

Supplementary Figure

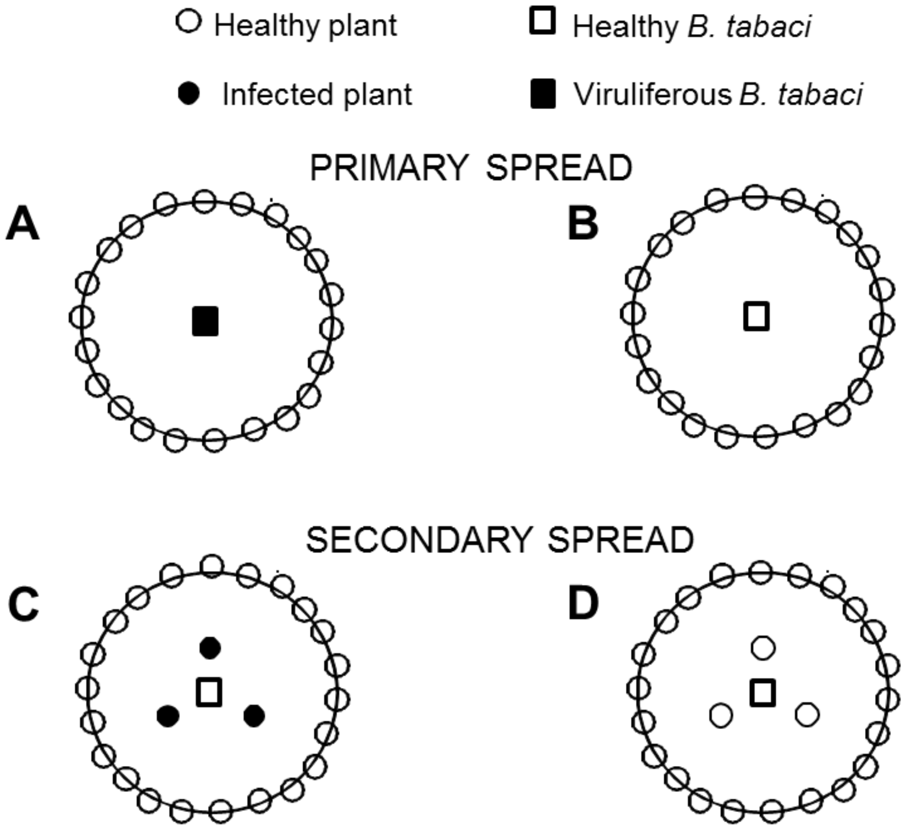

Supplementary Figure 1. Schematic representation of primary and secondary spread experimental design. For primary spread (A) of an isolate of the strain IL of Tomato yellow leaf curl virus (TYLCV-IL), viruliferous *Bemisia tabaci* adult individuals (15 whiteflies per test plant) were released in the centre of a circle (2 m diameter) of 22 healthy test plants. For secondary spread (C) from virus source plants, three TYLCV-IL-infected plants were placed forming a triangle (with 60 cm between each of the three plants) in the centre of a circle (2 m diameter) of 22 healthy test plants. Healthy *B. tabaci* adult individuals (30 whiteflies per test plant) were then released in the centre of the triangle of virus-source plants. Control experiment were conducted using healthy whiteflies for primary spread (B) and healthy source plants for secondary spread (D). Open and filled circles indicate healthy or TYLCV-infected plants, respectively. Open and filled squares indicate healthy or TYLCV-IL viruliferous whiteflies, respectively.
